# Screening of Compounds for Anti-tuberculosis Activity, and *in vitro* and *in vivo* Evaluation of Potential Candidates

**DOI:** 10.3389/fmicb.2021.658637

**Published:** 2021-06-30

**Authors:** Wei Zhou, Bing Yang, Yanyan Zou, Khaista Rahman, Xiaojian Cao, Yingying Lei, Ren Lai, Zhen F. Fu, Xi Chen, Gang Cao

**Affiliations:** ^1^State Key Laboratory of Agricultural Microbiology, Huazhong Agricultural University, Wuhan, China; ^2^College of Veterinary Medicine, Huazhong Agricultural University, Wuhan, China; ^3^Key Laboratory of Animal Models and Human Disease Mechanisms, Chinese Academy of Sciences and Yunnan Province, Kunming Institute of Zoology, Kunming, China; ^4^Bio-Medical Center, Huazhong Agricultural University, Wuhan, China; ^5^Cooperative Innovation Center for Sustainable Pig Production (CICSPPS), Huazhong Agricultural University, Wuhan, China

**Keywords:** *Mycobacterium tuberculosis*, orbifloxacin, drug resistance, DNA gyrase, molecular docking, tuberculosis, combination therapy

## Abstract

Tuberculosis (TB) is a debilitating infectious disease responsible for more than one million deaths per year. The emergence of drug-resistant TB poses an urgent need for the development of new anti-TB drugs. In this study, we screened a library of over 4,000 small molecules and found that orbifloxacin and the peptide AK15 possess significant bactericidal activity against *Mycobacterium tuberculosis* (*Mtb*) *in vitro*. Orbifloxacin also showed an effective ability on the clearance of intracellular *Mtb* and protect mice from a strong inflammatory response but not AK15. Moreover, we identified 17 nucleotide mutations responsible for orbifloxacin resistance by whole-genome sequencing. A critical point mutation (D94G) of the DNA gyrase (*gyrA*) gene was found to be the key role of resistance to orbifloxacin. The computational docking revealed that GyrA D94G point mutation can disrupt the orbifloxacin–protein gyrase interactions mediated by magnesium ion bridge. Overall, this study indicated the potential ability of orbifloxacin as an anti-tuberculosis drug, which can be used either alone or in combination with first-line antibiotics to achieve more effective therapy on TB.

## Introduction

Tuberculosis (TB), caused by *Mycobacterium tuberculosis* (*Mtb*), is a life-threatening infectious disease, which was responsible for 1.3 million deaths globally in 2020 [World Health Organization (WHO) (2020)]. The World Health Organization reported that approximately 23% (1.7 billion) of the world’s population has been latently infected by *Mtb*, and 1.5 million people develop active TB every year. The treatment regimen for TB mainly comprises antibiotics such as rifampicin (RIF), isoniazid (INH), ethambutol, pyrazinamide, and aminoglycosides (Fatma et al., 2020). Therapy with these antibiotics must be taken for 6 months to 2 years, which can result in the emergence of drug resistance and poor therapeutic outcomes (Adams et al., 2011; Akos et al., 2015; Castro et al., 2020). With the emergence of multidrug-resistant (MDR) and extensively drug-resistant (XDR) strains (Tao et al., 2017; Arthur et al., 2019), tackling the disease using currently available therapeutic regimens is a huge challenge (Koul et al., 2011; Huynh and Marais, 2019; Fuad et al., 2020). Therefore, there is a desperate need for novel, effective anti-TB drugs that can render the bacteria more susceptible to treatment.

Over the last few decades, numerous attempts have been made to develop drugs to combat drug-resistant strains of *Mtb* (Soutter et al., 2016; Jansen and Rhee, 2017; Mosaei et al., 2018). Genes essential for mycobacterial growth (Sabine et al., 2015; Zuniga et al., 2015), enzymes associated with fatty acid synthesis (Tiago et al., 2019), and DNA replication have been targeted for new treatment options (Aggarwal et al., 2017; Pradhan and Sinha, 2018). However, clinical investigations revealed that the main mechanisms of drug resistance in *Mtb* are gene mutations and biofilm formation (Poushali et al., 2021; Saba et al., 2021). Therefore, screening of natural products and their derivatives (Guzman et al., 2012), new small molecule inhibitors (Mishra et al., 2018), and FDA-approved drugs offers a new approach for the development of novel anti-TB drugs (Igarashi, 2017; Luo et al., 2017; Igarashi et al., 2018; Nadav et al., 2018). Virtual screening using docking programs has been used to identify potential drug molecules from a virtual library containing 461,397 compounds. Following this, the biological activity of the identified molecules has been evaluated *in vivo* in a zebrafish model (Adams et al., 2011; Takaki et al., 2012).

In recent years, quinolones (Kumar et al., 2009) have been widely used in clinical treatment because of their high efficiency and broad-spectrum antibacterial activity (Grossman et al., 2014; Peraman et al., 2016; Correia et al., 2017; Samiksha et al., 2019). A therapeutic regimen using moxifloxacin, a fluoroquinolone derivative, has been developed for the treatment of patients with MDR and XDR-TB (Huynh and Marais, 2019; Nunn et al., 2019). However, the mechanism of action and details of the drug–*Mtb* interactions remain elusive.

In this study, we screened a library of over 4,000 small molecules, including natural products, FDA-approved drugs, and peptides, for compounds exhibiting activity against *Mtb*. Orbifloxacin, the third generation of fluoroquinolone, was found to show potent anti-*Mtb* activity, both *in vitro* and *in vivo*, by targeting DNA gyrase subunit A (GyrA). Furthermore, we identified the mutations GyrA D94G and molecular mechanisms underlying orbifloxacin resistance.

## Materials and Methods

### Ethics Statements

This study was carried out in accordance with the standards set by the Administration of Animal Care and Usage for Research and the Ministry of Health of China. Animals were housed in a specific-pathogen-free environment at the laboratory animal center of Huazhong Agricultural University. All experiments were approved by the Committee on the Ethics of Animal Experiments of the College of Veterinary Medicine, Huazhong Agricultural University (HZAUMO-2019-038).

### Bacterial Strain and Culture Conditions

The *Mtb* H37Ra used in this study was obtained from the American Type Culture Collection (ATCC25177) and maintained in Difco Middlebrook 7H9 broth (Becton Dickinson), supplemented with 0.5% glycerol, 0.05% Tween 80, and 10% oleic acid albumin dextrose catalase (OADC, BD, United States) at 37°C. For bacterial culture and infection, all experiments were carried out in a biosafety level 2 laboratory following the appropriate biosafety standard operating procedures.

### Library of Drugs

A total of 4,476 compounds from the natural products library, NIH clinical library, FDA-approved library, spectrum collection library, CYX library, LXD library, and peptide libraries were purchased from the Shanghai Institute of Materia Medica of the Chinese Academy of Sciences and Kunming. The compounds were aliquoted into 96-well microplates at a stock concentration of 10 mM. For drug screening, these compounds were diluted 10-fold with phosphate-buffered saline (PBS) to evaluate their bioactivity and safety.

### Microplate Alamar Blue Assay

*Mycobacterium tuberculosis* H37Ra was cultured to mid-log phase, with an optical density (OD) of 0.6 (∼5 × 10^7^ colony-forming units [CFU]/ml) at 600 nm. One hundred microliters (5 × 10^4^ CFUs) of bacterial suspension was added to each well of 96-well microplates. Then, 10 μM of the compound was added to each well. DMSO was used as the negative control, and rifampicin (RIF) was used as the positive control. The plates were sealed and incubated at 37°C for 6 days. A 10% (v/v) solution of Alamar Blue was then added to each well. The anti-*Mtb* effect of each compound was determined based on the color change. The compound was considered to be inactive against *Mtb* if the color changed from blue to red.

### Determination of the Minimum Inhibitory Concentration of Selected Compounds

For minimum inhibitory concentration (MIC) analysis, 100 μl of the bacterial suspension was prepared as above and added to the wells of a 96-well plate. Ten microliters of twofold serial dilutions of screened drugs (from 0.012 to 12.8 μg/ml) and peptides (from 1 to 512 μg/ml) was added to the indicated well. Bacterial growth was assessed visually after culture for 1 week.

### Checkerboard Test of Antimicrobial Orbifloxacin Combinations

Checkerboard assays with *Mtb* H37Ra were used to assess the synergic activity of orbifloxacin in combination with first-line anti-TB drugs as described in previous studies (Bonapace et al., 2002; Bhusal et al., 2005). The activity of orbifloxacin was tested in combination with RIF, INH, streptomycin, kanamycin, ethambutol, and ethionamide. A total of 100 μl of the bacterial suspension was added to 96-well microplates as previously described. A serial twofold dilution of orbifloxacin, ranging from 1/8-fold MIC to fourfold MIC, was added to each row, and the other drugs, diluted from 1/8-fold MIC to fourfold MIC, were added to each column. Each combination was tested in triplicate for biological statistics. The plates were incubated at 37°C for 7 days, and bacterial growth was visually assessed. The fractional inhibitory concentration (FIC) index for each drug combination was calculated as follows:

$$\mathrm{FIC}\mathrm{index}\left(\mathrm{FICI}\right)=\mathrm{combination}\mathrm{MIC}A{/}_{A}+$$

$$\mathrm{combination}\mathrm{MIC}B{/}_{B}$$

The combination MIC is the concentration of a single drug in the mixture that can inhibit the growth of the bacteria. A or B is the MIC of the drug when used on its own. The FIC index values were interpreted as follows: FIC index ≤ 0.5, synergic effect; 0.5 < FIC index < 1, additive effect; 1 < FIC index < 2, indifferent effect; and FIC index ≥ 2, antagonistic effect.

### Cell Line Culture and Cytotoxic Assays

RAW264.7 (ATCC, TIB-71) cells were maintained in high-glucose Dulbecco’s modified Eagle’s medium (DMEM, Gibco), and THP-1 cells were cultured in RPMI-1640 medium supplemented with 10% fetal bovine serum (FBS, Gibco), 50 units/ml penicillin, and 50 μg/ml streptomycin (Gibco) at 37°C in a 5% CO_2_ incubator. For the cytotoxicity assay, 5 × 10^3^ RAW264.7 or THP-1 cells were seeded into 96-well microplates and incubated overnight. The cells were treated with a range of concentrations (from 0 to 64 μg/ml) of different drugs or peptides for 48 h, and it also applied to the 50% effective cytotoxic concentration value determination (CC_50_). Then, 10 μl of the MTS kit reagent solution was added. DMSO was used as the negative control. After 4 h of incubation, cell viability and cytotoxicity were measured using a CellTiter 96 AQ_*ueous*_ One Solution Cell Proliferation Assay (MTS, Promega, Cat# G3580), and the absorbance was recorded at 490 nm using a 96-well microplate reader. All cell experiments were conducted using no more than four generations of subculture after thawing of the cell stocks. A mycoplasma-free test was performed by using the myco-blue mycoplasma detector (D101-02, Vazyme, Nanjing, China).

### Drug Susceptibility Testing Against Intracellular *Mtb*

For *in vitro* antimicrobial activity studies, 2 × 10^5^ RAW264.7 cells were seeded into 12-well plates overnight and infected with H37Ra at a multiplicity of infection of 5 for 4 h at 37°C under a 5% CO_2_ atmosphere. Cells were washed three times with sterile PBS to stop infection. Fresh DMEM supplemented with 10% FBS, containing orbifloxacin (4 μg/ml), peptide (12.8 μg/ml), and a combination of orbifloxacin and peptide, was applied to each well. Kanamycin was used as a positive control against extracellular *Mtb*. After incubation for 48 h, the cells were washed with PBS and lysed with sterile 0.1% Tween 80. Cell lysates were diluted and plated on 7H11 agar plates. CFUs were enumerated after 3–4 weeks of incubation.

### Whole-Genome Sequencing of the Drug-Resistant Mutants

Drug resistance was induced in Mtb H37Ra by co-culturing it with orbifloxacin at a concentration of 1/4-fold to 10-fold MIC for three generations until the last generation showed a visible growth of Mtb in the presence of 10-fold MIC of orbifloxacin. Drug-resistant mutants were isolated from a single colony and grown on a 7H11 agar plate containing a 10-fold MIC concentration of orbifloxacin. Genomic DNA was extracted using the cetyltrimethylammonium bromide–phenol chloroform method. Briefly, bacterial pellets were re-suspended in GTE lysis buffer (50 mM glucose, 25 mM Tris, 10 mM EDTA; pH 8.0) containing lysozyme at a final concentration of 100 μg/ml and incubated at 37°C overnight. RNase A (10 μg/ml), 2% SDS solution, and protease K (10 μg/ml) were added, and the mixture was incubated at 56°C for 2 h. Chloroform–isopentanol and 75% ethanol were then used to isolate and purify the genomic DNA. The isolated DNA was quantified using a Nanodrop 2000 spectrophotometer (Thermo Fisher, United States). The DNA (2 μg) was fragmented by sonication and purified by gel extraction kit (Cat# D2500-02, OMEGA Bio-tek, United States). Fragmented DNA was repaired using the Hieff NGS Fast-Pace End Repair/dA-Tailing Module (Cat# 12608, YEASEN, United Kingdom) and ligated with a Y-adapter. Paired-end DNA libraries were constructed using a 14-cycle PCR amplification for Illumina next-generation sequencing (Hiseq 2000 platform). The quality of the DNA library was measured using Qubit 4 fluorometer (Invitrogen), and the DNA library was sent to Annoroad Gene Technology Company (Beijing) for high-throughput DNA sequencing.

### Determination of the Structure of the Ligand–Receptor Complex by Molecular Docking

The structure of orbifloxacin as a complex with *Mtb* GyrA was determined using Discovery Studio 2018 software based on the receptor–ligand interaction properties. The X-ray crystal structure of *Mtb* GyrA (PDB code: 5BS8) was obtained from the RCSB Protein Data Bank^1^. The 3D structure of orbifloxacin (PubChem ID: 60605) was downloaded from the open chemistry database PubChem^2^. The amino acid residue Asp94 of the wild-type protein was replaced with Gly94 to create a mutant GyrA crystal structure, using the Build and Edit Protein module of the software. The drug–protein binding model was predicted using the CDOCKER docking module, based on receptor–ligand interaction properties. The water molecules in the original crystal structure were removed prior to molecular docking. The binding site of the protein and orbifloxacin was found to be the active site cavity of GyrA, which is composed of A:Ala90, A:Asp94/Gly94, B:Gly459, B:Arg482, B:Thr500; B:Glu501, C:Arg128, and C:Ptr129. The default settings of CDOCKER were used. Docking results were analyzed and visualized using the “View Interactions” module in receptor–ligand interaction properties.

### Cloning of the Point Mutant and Wild-Type *gyrA* (Rv0006) Gene

Primers were designed based on sequences obtained from the National Center for Biotechnology Information (NCBI)^3^. The full-length of *gyrA* gene (Rv0006) amplified from the orbifloxacin-resistant strain and the wild-type strain was inserted into the plasmid, pMV261-GroEL, to obtain pMV261-GroEL-Mutant *gyrA* and pMV261-GroEL-WT *gyrA*, respectively. The recombinant plasmids were electroporated into *Mtb* H37Ra competent cells using an electric pulse machine for 20 ms, under a running program of 2.5 kV, 1,000 Ω, and 25 μF, and then plated onto Middlebrook 7H11 agar plates containing kanamycin (100 μg/ml) or orbifloxacin (five-fold MIC, 1 μg/ml). Single colonies were grown for MIC determination after 4 weeks of culture containing the antibiotic selection.

### Animal Experiments

C57BL/6 mice (female mice, 6−8 weeks old, 20 ± 2 g) were purchased from Beijing Vital River Laboratory Animal Technology and raised under specific pathogen-free conditions at the Laboratory Animal Center of Huazhong Agricultural University. All animals were randomly divided into three groups of 6–8 mice in each. Mice were infected intravenously with 0.2 ml *Mtb* H37Ra suspension at a dose of 5 × 10^6^ CFU/ml for 4 days. The infected mice were then orally administered orbifloxacin at a dose of 50 mg/kg per day. Isoniazid was used as a positive control at a dose of 12.5 mg/kg, and PBS was used as a negative control. The drugs were administered to the mice for 7 days post of infection. The mice were euthanized 18 days after infection. The lungs were used for histopathological and CFU analysis. A part of the left lung was fixed in 4% paraformaldehyde for 48 h, and tissues were embedded in paraffin to prepare sections for hematoxylin and eosin staining. Homogenates of lungs were plated onto Middlebrook 7H11 agar, containing OADC enrichment and BBL MGIT PENTA antibiotics (Cat# 245114, BD, United States), for bacterial burden enumeration. CFUs were counted after 3–4 weeks of incubation at 37°C.

### Cytokine Analysis

RNA was extracted from the lungs of mice, using the chloroform–isopropanol method. RNA was reverse transcribed using ReverTra Ace qPCR RT Master Mix, with a gDNA Remover Kit (Code No. FSQ-301, TOYOBO, Japan), according to the manufacturer’s instructions. SYBR Select Master Mix (Cat# 4472919, Thermo Fisher, United States) was used for the detection of murine tumor necrosis factor α (TNF-α), interleukin (IL) 4, IL-1β, IL-6, IL-10, and interferon β (IFN-β), and glyceraldehyde 3-phosphate dehydrogenase (GAPDH) was used as the housekeeping gene. Real-time PCR was run on a QuantStudio 6 real-time PCR system (ABI, United States), and fold change expression was calculated using the 2^−ΔΔ*Ct*^ method normalized to GAPDH (Emily et al., 2021).

### Data Availability

The raw data of the whole-genome sequence was submitted to the Sequence Read Archive (SRA) on the NCBI website (BioProject accession number: PRJNA661429).

### Statistical Analysis

Numerical data from three independent experiments were analyzed using GraphPad Prism 6.0 (La Jolla, CA, United States) and are shown as mean ± standard deviation or standard error of the mean. The significance of differences between the groups was evaluated using one-way ANOVA or Student’s *t*-test. Statistical differences were considered to be significant at *p* < 0.05; *p* values of <0.05, <0.01, <0.001, and <0.0001 are indicated as ^∗^, ^∗∗^, ^∗∗∗^, and ^****^,**** respectively.

## Results

### Screening the Small Molecule Library Against *Mtb*

**FIGURE 1:**
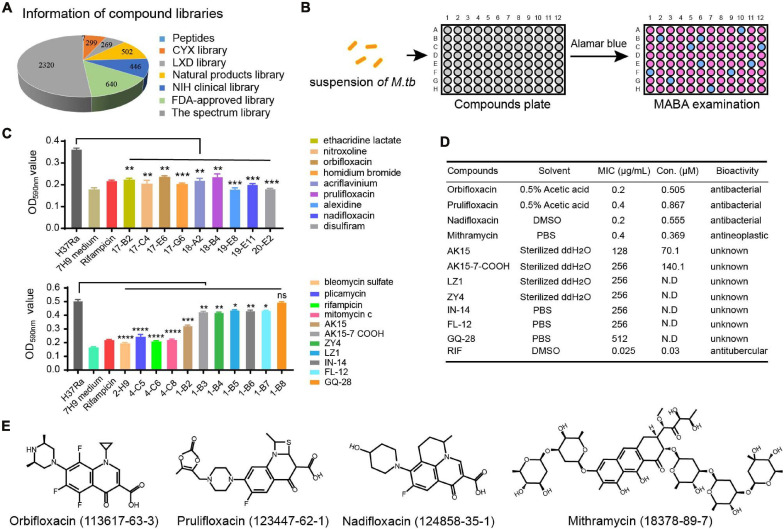
*In vitro* screening of drug libraries against *Mycobacterium tuberculosis* H37Ra. **(A)** The distribution of each group of compounds in the drug libraries. **(B)** Procedure for the drug screening: a defined concentration of each compound from the libraries was inoculated with a suspension of *Mtb* in 96-well microplates for 7 days, and the antibacterial effects were determined by Alamar Blue assay. **(C)** Graphical representation of the antibacterial effects of selected compounds. **(D)** Determination of MIC of selected compounds and peptides, as described in the chart. Rifampicin (RIF) was used as a positive control. These results are presented as mean ± SD from triplicates. Two-tailed unpaired Student’s *t*-test was used for analysis: N.D., not detectable; *p < 0.05; **p < 0.01; ***p < 0.001; ****p < 0.0001. **(E)** Chemical structures of orbifloxacin, prulifloxacin, nadifloxacin, and mithramycin.

A library of compounds, including 502 small molecules of natural origin and their derivatives, 299 and 269 bioactive compounds isolated from traditional Chinese medicine recipes, seven small peptides, and 640 FDA-approved drugs were screened for their anti-TB activity, based on their effect on the growth of *Mtb in vitro* (Figures 1A,B). The large-scale screening of the compounds in microplates (Supplementary Figure 1A) showed that antibiotics including orbifloxacin, prulifloxacin, nadifloxacin, disulfiram, and mithramycin, and antimicrobial peptides including AK15, AK15-7COOH, LZ1, and ZY4 exhibited significant anti-*Mtb* activity (Figure 1C). Amongst the compounds tested, the fluoroquinolones (orbifloxacin, prulifloxacin, and nadifloxacin) and mithramycin were found to show the best anti-TB activity, exhibiting MICs of 0.2–0.4 μg/ml. The peptide AK15 also presented favorable activity against *Mtb* with a MIC of 128 μg/ml, and the remaining peptide (AK15-7COOH, LZ1, and ZY4) showed activity against *Mtb* with MIC of 256 μg/ml (Figure 1D). These data indicated that the drugs and peptides could have a potential antimicrobial activity on *Mtb*. The structures of the two bioactive peptides, AK15 and 15AK-7COOH, are shown in Supplementary Figure 1B. The chemical structures of the small molecules were obtained from the PubChem database (see text footnote 2) and shown in Figure 1E.

### Intracellular Bactericidal Effect of Orbifloxacin

**FIGURE 2:**
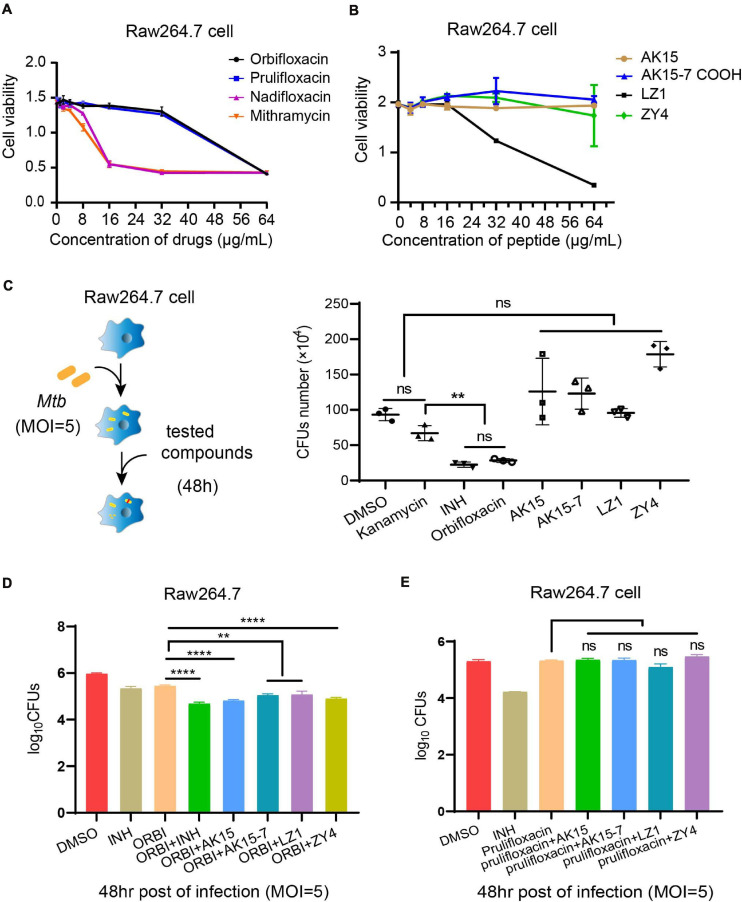
Cytotoxicity detection and intracellular anti-*Mycobacterium tuberculosis* activities of selected drugs. **(A,B)** A cell proliferation assay was used to detect the toxicity of orbifloxacin, prulifloxacin, nadifloxacin, mithramycin, and the peptides on RAW264.7 macrophages. **(C)** Procedure for intracellular drug susceptibility test in RAW264.7 cells and CFU enumeration of *Mtb* in murine macrophages treated with individual drugs for 48 h post infection. **(D)** Combination effects of orbifloxacin with the peptides against *Mtb in vitro*. **(E)** Combination effects of prulifloxacin with the peptides against *Mtb in vitro*. These experiments were performed in duplicate and analyzed using multiple comparison test in GraphPad Prism: ns, not significant; **p < 0.01; ****p < 0.0001 (mean ± SD from triplicates).

The cytotoxicity of the selected antibiotics (orbifloxacin, prulifloxacin, nadifloxacin, and mithramycin) and peptides (AK15, AK15-7COOH, LZ1, and ZY4) was tested using the MTS method. A decrease in OD_490 nm_ indicated cytotoxicity toward the RAW264.7 cell. We found that orbifloxacin and prulifloxacin were not cytotoxic at a concentration of 8 μg/ml, whereas nadifloxacin and mithramycin were cytotoxic at 16 μg/ml to RAW264.7 cells (Figure 2A), but showed distinct cytotoxicity toward THP-1 cells even at low concentrations (Supplementary Figure 2A). Orbifloxacin also displayed a very high safety cytotoxic concentration to Raw264.7 cell with 115.42 μM (MIC = 0.51 μM) (Supplementary Figure 3). The peptides AK15, AK15-7COOH, and ZY4 did not show any cytotoxicity up to 64 μg/ml; the maximum safe concentration of LZ1 was found to be 16 μg/ml (Figure 2B).

Next, we examined the effects of these drugs on the clearance of intracellular *Mtb* in macrophages by CFU assay (Figure 2C). We found that the number of CFUs from orbifloxacin-treated cells was significantly lower than that in the DMSO-treated control. Notably, there were no significant differences between the CFUs observed in the orbifloxacin compared with the isoniazid group. A significant difference was observed between the orbifloxacin and kanamycin (control for extracellular *Mtb* inhibition) groups. A combination of orbifloxacin with INH produced a significant decrease in the number of CFUs compared with treatment with either drug alone (Figure 2D). The peptides AK15, AK15-7COOH, LZ1, and ZY4 did not affect the clearance of intracellular *Mtb*, despite their *in vitro* efficacy (Figure 2C). Interestingly, a combination of these peptides with orbifloxacin significantly enhanced the effects of orbifloxacin in clearing intracellular *Mtb* (Figure 2D). However, a similar effect was not observed when they were used in combination with prulifloxacin (Figure 2E). Furthermore, we evaluated the activity of orbifloxacin in combination with first-line antibiotics (rifampicin, isoniazid, and ethambutol) and found that a combination of these drugs has four times more potent bactericidal activity than any of the drugs alone (Supplementary Figure 2B).

### *In vivo* Activity of Orbifloxacin

**FIGURE 3:**
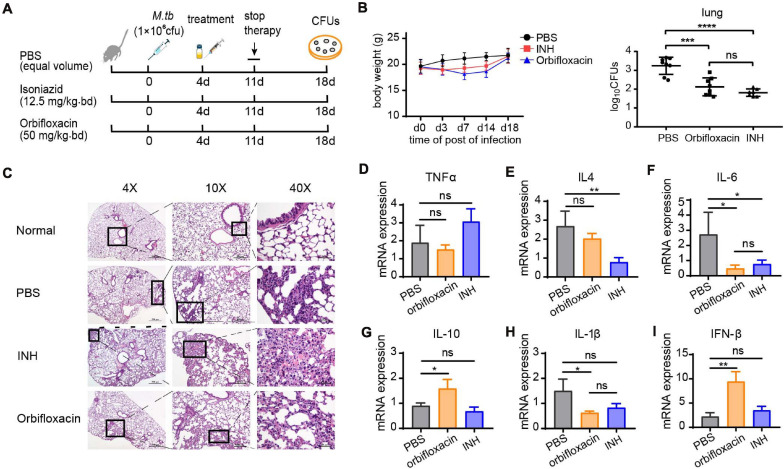
*In vivo* anti-*Mycobacterium tuberculosis* effects of orbifloxacin in a mouse model. **(A)** Schematic representation of the procedure for the *in vivo* assay in C57BL/6 mice (*n* = 7 or 8 in each group). **(B)** Changes in body weight were observed, and the bacterial burden in the lungs of the infected mice was determined by plating the organ lysate on the 7H11 plates after treatment with orbifloxacin or PBS for 7 days. Data are presented as mean ± SD. **(C)** Hematoxylin and eosin staining of lung sections of representative *Mtb-*infected mice from the drug-treated group and control group. **(D–I)** The expression of various cytokine genes was determined by quantitative PCR from the whole lung homogenates of mice from the drug-treated and control groups. ns, not significant; *p < 0.05; **p < 0.01; ***p < 0.001; ****p < 0.0001.

To further validate the anti-*Mtb* effect of orbifloxacin *in vivo*, we infected C57BL/6 mice with *Mtb* H37Ra and administered the drugs 4 days after infection for a total of 7 days. The mice were then sacrificed on day 18 postinfection for histopathological and bacterial burden analysis (Figure 3A). The mice in the orbifloxacin and INH groups (isoniazid used as a drug positive control) showed a slight weight loss (Figure 3B), with a significant reduction in the number of CFUs relative to that in the PBS control. There was no significant difference between the groups treated with orbifloxacin and INH (Figure 3B). Histopathological examination of lung tissues revealed that PBS-treated mice showed moderately higher infiltration of inflammatory cells, such as macrophages and lymphocytes, than mice treated with the drugs (Figure 3C). In PBS-treated mice, thickening of the alveolar wall, narrowing of the alveolar space, and the buildup of some red blood cells in the cavity were observed in the lungs. In contrast, the lesions were much milder and fewer macrophages were present in the alveolar cavity of the mice treated with orbifloxacin (Figure 3C). We also analyzed cytokine expression levels in the lungs of the mice. IL-6 and IL-1β expression were significantly lower in the orbifloxacin-treated group than in the PBS-treated group (Figures 3F,H). There were no significant differences in the expression of TNF-α in any of the groups (Figure 3D). The expression of IL-4, the representative cytokine of Th2 response showed slightly decrease in orbifloxacin treated mice (Figure 3E). IL-10 and IFN-β expression were significantly higher in the orbifloxacin-treated group than in the INH- or PBS-treated groups (Figures 3G,I), suggesting that orbifloxacin can protect mice from a strong inflammatory response. Overall, our results show that orbifloxacin exhibits strong *in vivo* anti-*Mtb* activity and could be considered as a potential drug for the treatment of TB.

### GyrA D94G Mutation Is Attributed to Orbifloxacin Resistance in *Mtb*

**FIGURE 4:**
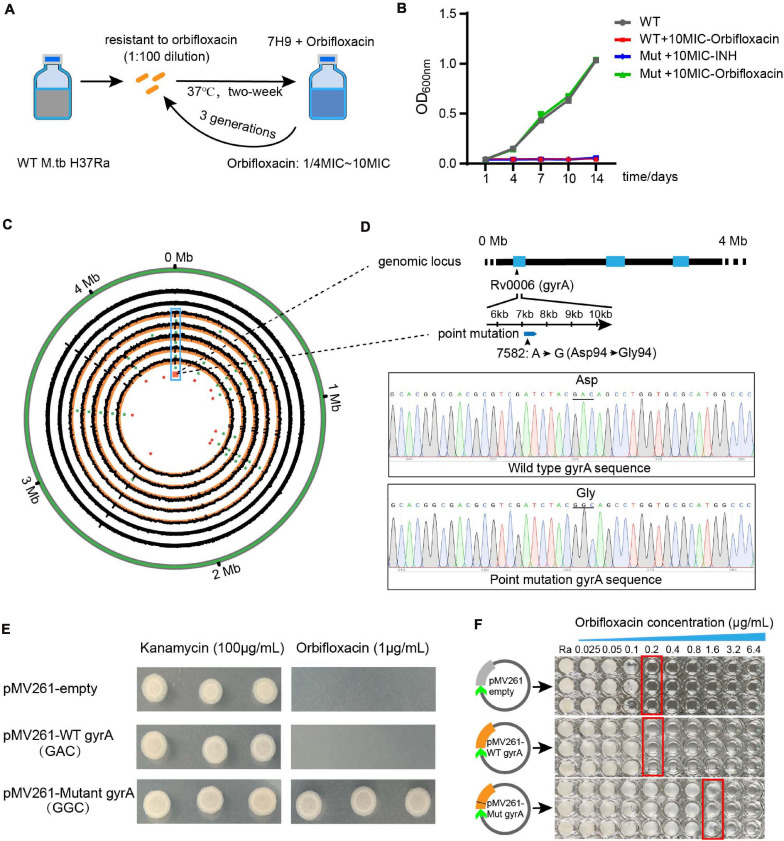
Whole-genome sequencing analysis of the orbifloxacin-resistant strain. **(A)** Wild-type *Mtb* H37Ra was treated with orbifloxacin at concentrations of 1/4-fold MIC to 10-fold MIC to induce drug resistance. **(B)** The acquired orbifloxacin resistance of the mutated strain was confirmed by comparing its growth on 7H9 medium, supplemented with 10-fold MIC of orbifloxacin, with that of the wild-type strain. **(C)** Circos plot represents the whole-genome sequence of the wild-type strain and the five orbifloxacin-resistant strains. **(D)** The point mutation of GAC to GGC in the Rv0006 gene (*gyrA*) is displayed as confirmed by Sanger sequencing. **(E)** Over-expression of the wild-type *gyrA* and mutant *gyrA* gene in wild-type *Mtb* and the growth of bacteria in the presence of 1 μg/ml of orbifloxacin. pMV261-empty and pMV261-WT *gyrA* were used as controls. **(F)** MIC of orbifloxacin in the *Mtb* strain expressing mutant *gyrA* [obtained from the plates in panel **(D)**].

To understand the cause of orbifloxacin resistance in *Mtb*, we induced the generation of orbifloxacin-resistant in an *Mtb* strain and confirmed the resistance using a MIC assay (Figure 4A). Because combination therapy using orbifloxacin and INH enhances the bactericidal action, we aimed to detect any potential cross-resistance to INH by culturing orbifloxacin-resistant strains of *Mtb* in the presence of INH. These mutated strains did not exhibit resistance to INH (Figure 4B). Next, five colonies of orbifloxacin-resistant *Mtb* were subjected to whole-genome sequencing, and 17 single nucleotide point mutations were observed, compared with the wild-type strain ATCC25177 (Figure 4C, circular map). Notably, mutations in *gyrA* were observed in all five resistant strains. Thus, we hypothesized that the *gyrA* gene might be the key to understanding orbifloxacin resistance on *Mtb*. Therefore, we analyzed the gene sequence of *gyr*A and found a single point mutation at the 94th amino acid residue, where the aspartic acid was replaced by glycine (GyrA D94G) (Figure 4D).

To confirm whether this point mutation is responsible for orbifloxacin resistance, we introduced a point mutation of D94 to G94 in the *gyrA* gene into wild-type *Mtb* as above. We found that the mutant *Mtb* strain could grow on a Middlebrook 7H11 agar plate supplemented with a fivefold MIC concentration of orbifloxacin (1 μg/ml), whereas *Mtb* transfected with a plasmid control or with a wild-type *gyrA* gene could not (Figure 4E). To further confirm the resistance, we grew a single colony of *Mtb* containing the mutation in *gyrA* in a 96-well plate in the presence of orbifloxacin and found that the MIC increased from 0.2 to 3.2 μg/ml, which displayed a stronger resistance activity to orbifloxacin (Figure 4F). These data suggest that the *gyrA* gene of *Mtb* is the target of orbifloxacin and that the *gyrA* D94G mutation may be responsible for resistance to orbifloxacin.

### Analysis of Ligand–Receptor Interactions by Structural Docking

**FIGURE 5:**
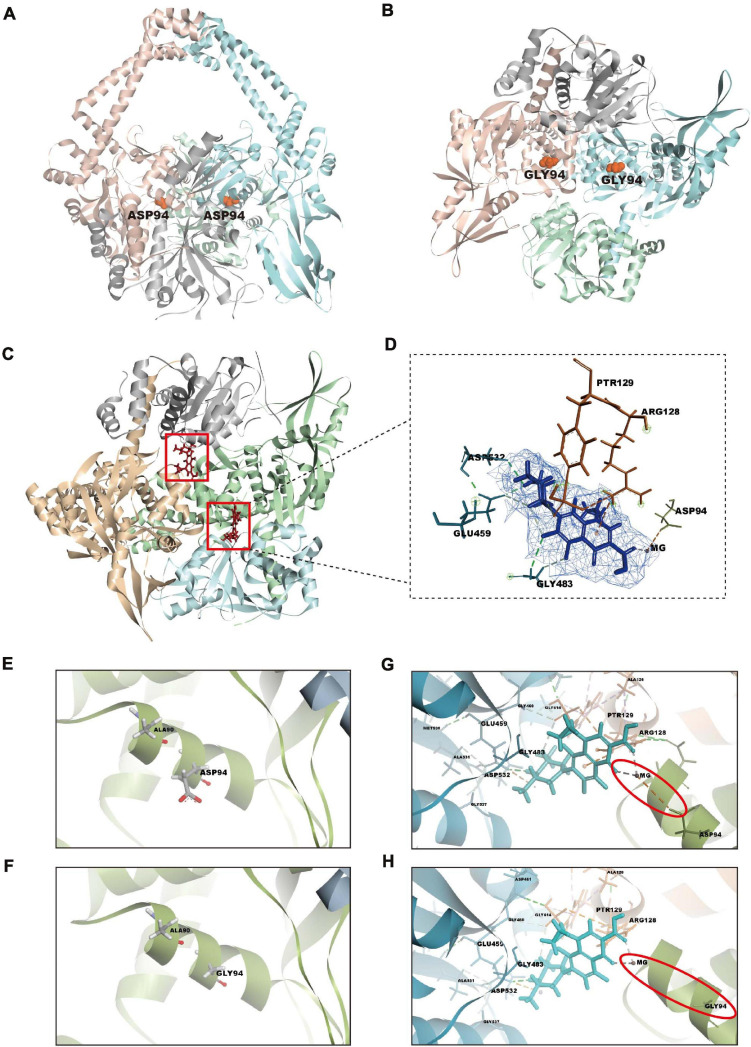
Structural analysis of the GyrA-orbifloxacin complex and comparison of the binding of orbifloxacin to GyrA wild-type and GyrA D94G. **(A,B)** Overall structure of wild-type **(A)** and remodeled mutant gyrA protein **(B)**. *gyrA*^ D94G^ was created by replacing the 94th amino acid residue, Asp, with Gly in GyrA wild-type. **(C)** A cartoon representation of the GyrA wild-type–orbifloxacin complex. Orbifloxacin binding is mainly controlled by residues in the active site cavity of GyrA wild-type. **(D)** The detailed interactions and key contact sites of the complex. The residues that interact with orbifloxacin are marked. **(E,F)** Amino acid changes in GyrA D94G compared with GyrA wild-type. The side chains of the 94th amino acid residue in GyrA wild-type and GyrA D94G are labeled. **(G,H)** Comparison of binding interactions between GyrA wild-type–orbifloxacin and GyrA D94G–orbifloxacin. Magnesium ion-bridged interaction between the hydroxyl oxygen of Asp94 in gyrA with the fourth and sixth oxygen of orbifloxacin. This interaction in wild-type gyrA is disrupted by the D94G mutation.

To explore the molecular mechanism of action of orbifloxacin through its action on *gyrA*, we first modeled the crystal structure of the mutant gyrase subunit A, using Discovery Studio 2018 (Figure 5B), based on the structure of the wild-type *Mtb* gyrase (Figure 5A). Subsequently, the binding patterns of orbifloxacin with wild-type and mutant gyrase were modeled using the CDOCKER docking module, based on the receptor–ligand interaction properties. Orbifloxacin was seen to occupy the active site of the wild-type protein GyrA (Figure 5C). Orbifloxacin binding was found to be controlled by the Asp94, Arg128, Ptr129, Gly483, Glu459, and Asp532 residues in the binding site (Figure 5D). Ptr129 and Glu459 played an important role in this binding, forming hydrogen bonds with orbifloxacin. A favorable π-cation interaction between the benzene ring of orbifloxacin and the backbone hydrogen nitride of Arg128 was also predicted by the software. In addition, a single active site magnesium ion bridged the hydroxyl oxygen of Asp94 with the fourth and sixth oxygens of orbifloxacin. However, due to the difference between the side chains of Asp and Gly (Figures 5E,F), the corresponding favorable interaction was disrupted in the mutant protein GyrA, the details of which are depicted in Figures 5G,H. Our data suggest that D94G impairs the binding of orbifloxacin with protein GyrA, which could explain the mechanism of resistance to orbifloxacin in *Mtb*.

## Discussion

Tuberculosis is a white plague that kills millions of people every year. Several drugs have been developed and used clinically for TB for decades (Yuan and Sampson, 2018). Conventional drugs take more than half a year to effectively treat the infection; however, many patients are unable to complete the course of treatment due to its serious side effects. In addition, long-term use of these antibiotics has led to the emergence of drug resistance, which poses a huge threat to human life. MDR and XDR *Mtb* infections have been reported worldwide (Seung et al., 2015; Sander et al., 2020). In this study, we found that orbifloxacin had high anti-*Mtb* bioactivity *in vitro* with a low MIC of 0.2 μg/ml and protected the mice from infection *in vivo*. Interestingly, the use of orbifloxacin in combination with INH and RIF exhibited more potent bactericidal activity against *Mtb in vitro*. Such combination therapy not only reduces the required dose and the frequency of drug administration but also reduces the duration of treatment, thus reducing side effects and the risk of developing drug resistance. Therefore, combination therapy involving orbifloxacin could be further evaluated as a new treatment regimen for TB.

Antibiotics without cross-resistance, as well as combination therapies, have been assessed in clinical trials for the treatment of patients with INH-resistant TB (Hu et al., 2017; Tweed et al., 2019). In the current study, we found that a mutation of the 94th amino acid of protein GyrA is likely to be responsible for resistance to orbifloxacin, without affecting INH susceptibility because of the different target on the mechanism of action such as *katG* gene or inhA (Arash et al., 2019; Flentie et al., 2019). Although mutations in the *gyrA* gene have been linked to fluoroquinolone resistance in *Mtb* such as moxifloxacin (Aldred et al., 2016; Blower et al., 2016; Petrella et al., 2019), the detailed mechanism of *Mtb* resistance to orbifloxacin has not been elucidated. With the low cure rate and high recurrence rate of drug-resistant TB, the discovery of orbifloxacin targets and elucidation of mechanisms of orbifloxacin resistance facilitate effective therapy for no cross drug-resistant TB and may offer a good basis for the rational design of treatment of drug-resistant TB. Data from computational studies in Figure 5 demonstrate the interactions of *Mtb* with orbifloxacin *via* a magnesium bridge from the Asp94 in the active site. This interaction was also predicted to be the mechanism underlying fluoroquinolone resistance (Yu et al., 2017; Rhastin et al., 2020). Understanding this mechanism provides a promising strategy for the study and development of synthetic drugs using orbifloxacin and its derivatives to face drug resistance challenges.

In this study, we screened a library of small molecules and peptides and identified orbifloxacin as a potential drug candidate for anti-TB treatment. No cross-resistance was observed between isoniazid and orbifloxacin because of the different targets; this drug might be useful for the treatment or combination therapy of INH-resistant TB. Moreover, using an orbifloxacin–GyrA enzyme model, we found that a single mutation in *gyrA* (Gyrase subunit A D94G) is able to reduce the interaction of orbifloxacin with protein GyrA. These data can enable the development of orbifloxacin derivatives that can bind to this mutant site for high efficiency against drug-resistant *Mtb* and thus be used in the treatment of fluoroquinolone-resistant *Mtb*. In conclusion, our study identified a potential anti-TB drug candidate and elucidated its mechanism of action, which could contribute to the rational design of more potent anti-TB drugs and help combat MDR and XDR-TB.

## Data Availability Statement

The datasets presented in this study can be found in online repositories. The names of the repository/repositories and accession number(s) can be found below: https://www.ncbi.nlm.nih.gov/, PRJNA661429.

## Ethics Statement

The animal study was reviewed and approved by the committee on the ethics of animal experiments of the college of veterinary medicine, Huazhong Agricultural University (HZAUMO-2019-038).

## Author Contributions

WZ, BY, YZ, KR, XC, and GC: data curation. ZF, XC, and GC: funding acquisition. WZ and BY: investigation and writing original draft. WZ, BY, and GC: project administration. RL: resources. ZF and GC: supervision. WZ: validation. YZ, KR, XJC, YL, XC, and GC: writing review and editing. All authors contributed to the article and approved the submitted version.

## Conflict of Interest

The authors declare that the research was conducted in the absence of any commercial or financial relationships that could be construed as a potential conflict of interest.

## Footnotes

1 https://www.rcsb.org

2 https://pubchem.ncbi.nlm.nih.gov

3 https://www.ncbi.nlm.nih.gov/gene

## Supplementary Material

Supplementary Table 1 Primers used for qRT-PCR.

## Abbreviations

TB: tuberculosis
*Mtb*: *Mycobacterium tuberculosis*
RIF: rifampicin
INH: isoniazid
MDR: multiple drug-resistant
XDR: extensively drug-resistant
*gyrA*: DNA gyrase subunit A
MIC: minimum inhibitory concentration
CFU: colony-forming unit
PBS: phosphate-buffered saline
FIC: fractional inhibitory concentration.
