# Supplementary material for: Screening of Compounds for Anti-tuberculosis Activity, and in vitro and in vivo Evaluation of Potential Candidates
Source: Front Microbiol. 2021 Jun 30;12:658637. doi: 10.3389/fmicb.2021.658637 (PMC8278749; doi:10.3389/fmicb.2021.658637)
Supplement: Supplementary file 2 [file Data_Sheet_2.docx]

Supplementary Material

# Supplementary Figures and Tables

## Supplementary Figures


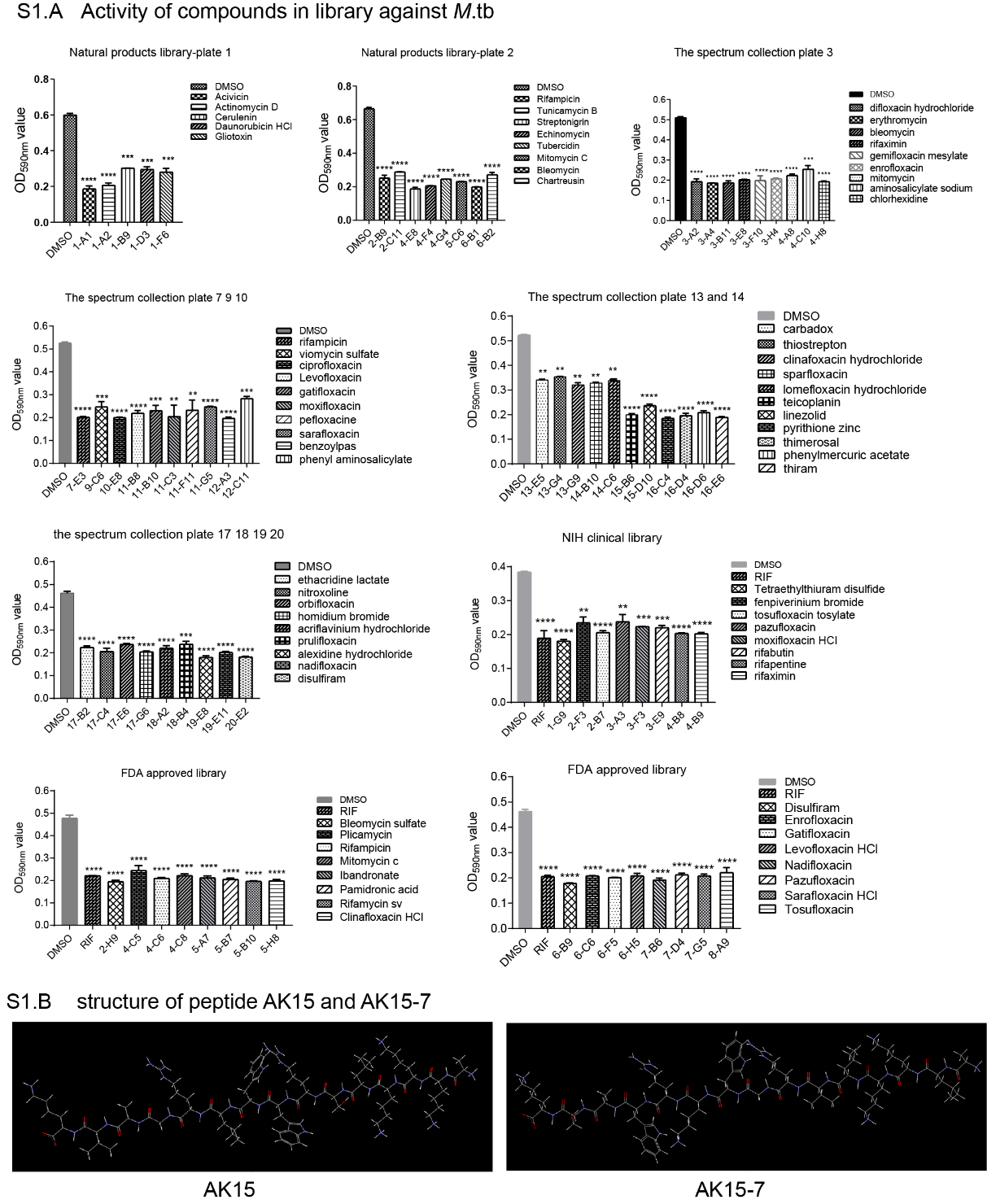


**Supplementary Figure 1.** **(A)** Results of anti-*mycobacterium tuberculosis* compounds screening in all of the library. **(B)** the structure of peptides AK15 and AK15-7.


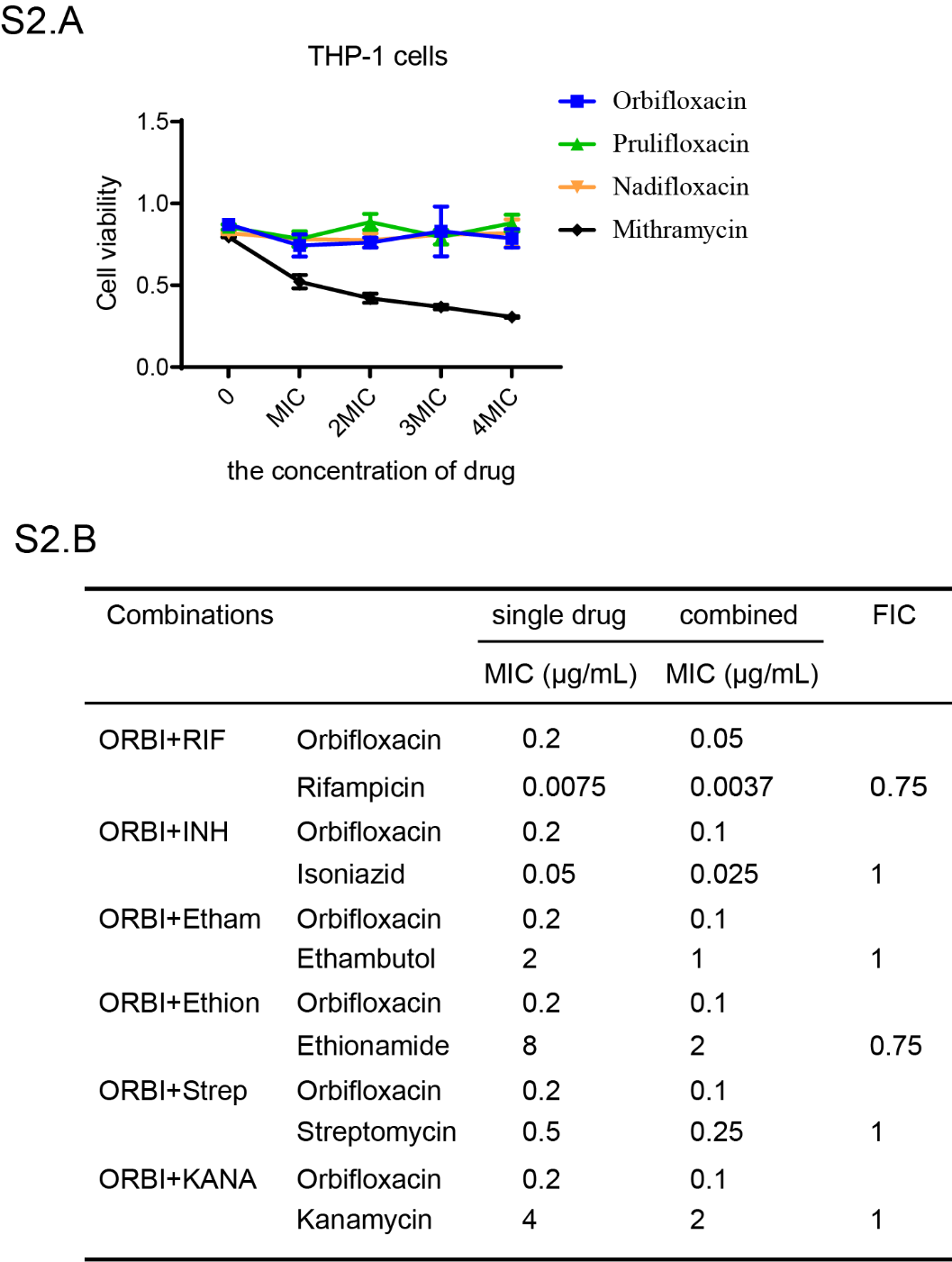


**Supplementary Figure 2.** **(A)** Cell proliferation assay were used to detect the toxicity of orbifloxacin, prulifloxacin, nadifloxacin and mithramycin on THP-1 cells. **(B)** The combined antibiotic susceptibility tests of orbifloxacin with the first-line drugs against *mycobacterium tuberculosis in vitro.*

## Supplementary tables

**Table S1. List of primers of qRT-PCR**

**Gene name sequence (5’ to 3’)**

**m-TNF-α forward: TCACTGGAGCCTCGAATGTC**

**reverse: GTGAGGAAGGCTGTGCATTG**

**m-IL-4 forward: ATGGAGCTGCAGAGACTCTT**

**reverse: AAAGCATGGTGGCTCAGTAC**

**m-IL-6 forward: TCCAGTTGCCTTCTTGGGAC**

**reverse: GTGTAATTAAGCCTCCGACTTG**

**m-IL-10 forward: TGTCCAGCTGGTCCTTTGTT**

**reverse: ACTGCACCCACTTCCCAGT**

**m-IL-1β forward: AGAAACAGTCCAGCCCATAC**

**reverse: CTGGTACATCAGCACCTCAC**

**m-IFN-β forward: CCTATGGAGATGACGGAGAAGA**

**reverse: AGTGGAGAGCAGTTGAGGACAT**
